# Dried Blood Spot Method Development and Clinical Validation for the Analysis of Elexacaftor, Elexacaftor-M23, Tezacaftor, Tezacaftor-M1, Ivacaftor, Ivacaftor Carboxylate, and Hydroxymethyl Ivacaftor Using LC-MS/MS

**DOI:** 10.1097/FTD.0000000000001231

**Published:** 2024-08-27

**Authors:** Steffie E. M. Vonk, Marloes van der Meer-Vos, Renate Kos, Anne H. Neerincx, Suzanne W. J. Terheggen-Lagro, Josje Altenburg, Anke H. Maitland-van der Zee, Ron A. A. Mathôt, E. Marleen Kemper

**Affiliations:** * Department of Hospital Pharmacy and Clinical Pharmacology, Amsterdam UMC, University of Amsterdam, Amsterdam, the Netherlands;; † Department of Pulmonary Medicine, Amsterdam UMC, University of Amsterdam, Amsterdam, the Netherlands;; ‡ Department of Pediatric Pulmonology and Allergy, Amsterdam UMC, University of Amsterdam, Emma Children's Hospital, Amsterdam, the Netherlands; and; § Department of Vascular Medicine, Amsterdam UMC, University of Amsterdam, Amsterdam, the Netherlands.

**Keywords:** elexacaftor–tezacaftor–ivacaftor, cystic fibrosis, LC-MS/MS, therapeutic drug monitoring, dried blood spot

## Abstract

**Background:**

The highly effective cystic fibrosis transmembrane conductance regulator (CFTR) modulator, elexacaftor–tezacaftor–ivacaftor, is now widely being used by people with cystic fibrosis. However, few independent studies have detailed the pharmacokinetics (PK) of CFTR modulators. Blood collection by venipuncture is the gold standard for PK measurements, but it is invasive. The aim of this study was to develop and clinically validate a quantification method for elexacaftor, tezacaftor, ivacaftor, and their main metabolites in dried blood spots (DBSs) using liquid chromatography with tandem mass spectrometry.

**Methods:**

Linearity, accuracy, precision, stability, hematocrit (Hct), spot-to-spot carryover, spot volume, and extraction efficiency were validated in DBS for all analytes. The clinical validation of elexacaftor–tezacaftor–ivacaftor in patients was performed by comparing 21 DBS samples with matched plasma samples.

**Results:**

The preset requirements for linearity, within-run and between-run accuracy, precision, Hct, spot volume, and extraction efficiency were met. Puncher carryover was observed and resolved by punching 3 blanks after each sample. The samples remained stable and showed no notable degradation across the tested temperatures and time intervals. Corrected DBS values with the Passing–Bablok regression equation showed good agreement in Bland–Altman plots, and acceptance values were within 20% of the mean for a minimum of 67% of the repeats, according to the EMA guidelines.

**Conclusions:**

A quantification method for the analysis of elexacaftor, tezacaftor, ivacaftor, and their main metabolites was developed and clinically validated in DBS. This method could be valuable in both clinical care and research to address unanswered PK questions regarding CFTR modulators.

## Abstract 2

Supplemental Digital Content is Available in the Text.

## INTRODUCTION

The landscape of cystic fibrosis (CF) is changing, as the highly effective CF transmembrane conductance regulator (CFTR) modulating drug, elexacaftor–tezacaftor–ivacaftor (ETI), is now available for people with CF (pwCF) with at least 1 F508del mutation. A defect in CFTR results in chloride channel dysfunction and causes CF, an autosomal recessive disease. Until recently, supportive and symptomatic care was the only available form of CF treatment.^1^ However, new treatments that address the fundamental cause of the disease, modulators of CFTR, are currently widely used. The FDA and EMA have approved the following drugs for the treatment of CF: Ivacaftor (Kalydeco), lumacaftor–ivacaftor (Orkambi), tezacaftor–ivacaftor (Symdeko/Symkevi), and the most potent and most recently approved combination drug ETI (Trikafta/Kaftrio).

Tezacaftor–ivacaftor was available for fewer mutations and showed limited clinical efficacy: Only 20%–30% of pwCF responded to these therapies.^2,3^ Currently, higher response rates have been observed with the newest triple therapy.^4,5^ In a recent interim analysis of real-world data, a mean absolute change in percentage predicted forced expiratory volume in 1 second (ppFEV1) from baseline of +8.2% (95% CI, 8.0–8.4) after 1 year, which persisted after 2 years of treatment with a change of +8.9% (95% CI, 8.7–9.1). Similarly, other efficacy parameters, such as pulmonary exacerbations, hospitalizations, positive bacterial cultures, and BMI improved significantly.^6^ The safety profile in clinical trials was acceptable, with most adverse effects being mild-to-moderate. However, in real-world studies, a considerable number of nonresponders and pwCF experience side effects that sometimes force them to discontinue treatment or lower the dose.^7–9^

Differences in pharmacological efficacy and safety responses may be driven by differences in drug exposure. However, few independent studies have detailed the pharmacokinetics (PK) of CFTR modulators. Moreover, little is known regarding the steady-state plasma concentrations or therapeutic ranges of these agents. Thus far, PK information is available only for registration documentation.^10–13^ Knowledge of PK may provide further insights into the exposure–response relationships and interpatient variability of CFTR-modulating drugs. This knowledge may lead to a better understanding of the efficacy and safety of drugs and aid in the optimal treatment of pwCF. Furthermore, cytochrome P4503A4 significantly metabolizes all components of ETI, rendering them susceptible to potential drug–drug interactions when administered with comedications frequently prescribed for pwCF, such as azole antifungals.^14^ Hence, the measurement of drug concentrations is a valuable resource in certain scenarios. Blood collected via venipuncture is the gold standard for PK measurements. However, venipuncture is an invasive sampling procedure, particularly in children. Therefore, we aimed to clinically validate the determination of CFTR-modulating drugs in dried blood spots (DBSs), which are less invasive. This is especially advantageous when several samples are required during 1 dosing interval or when patients are not admitted to the hospital, because DBS sampling can also be performed at home.^15^ Second, DBS requires smaller blood volumes than plasma sampling, which is advantageous, especially in the pediatric population, where obtaining larger blood volumes can be challenging.^15^ Third, DBS facilitates remote monitoring because the samples show excellent stability during storage and transport. The dried paper protected the analytes from degradation, providing an opportunity for low-cost remote sampling and transportation.^15^ This is particularly relevant in the context of expanding telemedicine, which is currently of interest to CF care, as pwCF have better outcomes and visit the hospital less frequently.^16^ The largest limitation of DBS sampling is the risk of discarding samples that cannot be used because of multiple drops of blood on 1 DBS spot, spots that overlap, or spots that are too small for punching. Careful patient training is essential.^17^

**FIGURE 1.**
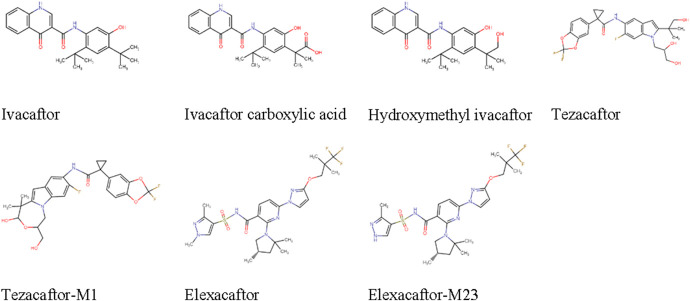
Chemical structures of ivacaftor, ivacaftor carboxylic acid, hydroxymethyl ivacaftor, tezacaftor, tezacaftor-M1, elexacaftor, and elexacaftor-M23.

In a previous study, we validated the quantification of ivacaftor, its metabolites, lumacaftor, and tezacaftor (Fig. 1) in plasma and sputum using chromatography-tandem mass spectrometry (LC-MS/MS).^18^ In this study, we aimed to extend this method and validate assessment of the following compounds in plasma: Elexacaftor, its active metabolite elexacaftor-M23, and the active metabolite of tezacaftor, tezacaftor-M1 (Fig. 1). Furthermore, this method was validated for the assessment of all compounds in DBS. Finally, the DBS sampling method was clinically validated in pwCF by using ETI.

## MATERIALS AND METHODS

### Standards, Reagents, and Chemicals

The reference standards ivacaftor, hydroxymethyl ivacaftor, and elexacaftor and the internal standards ivacaftor-D9 (plasma), elexacaftor-D3, and tezacaftor-D4 were purchased from Toronto Research Chemicals (TRC, Toronto, ON, Canada). Ivacaftor carboxylate was purchased from Clearsynth (Mumbai, India). Elexacaftor-M23 and tezacaftor-M1 were purchased from TLC Pharmaceutical Standards (Newmarket, ON, Canada). Tezacaftor was purchased from Chiron (Essex, United Kingdom). The IS ivacaftor-13C6 (not used) were purchased from Alsachim (Illkirch-Graffenstaden, France) and ivacaftor-D19 (not used) from Santa Cruz Biotechnology (Heidelberg, Germany), respectively. Ultrapure water was purified and deionized using a Purelab option DV-25 (Elga, High Wycombe, United Kingdom). Methanol (MeOH, hypergrade for LC-MS), acetonitrile (ACN, hypergrade for LC-MS), and formic acid were purchased from Merck Chemicals (Amsterdam, the Netherlands). Ammonium formate (MS grade) was purchased from Sigma-Aldrich (Steinheim, Germany). Drug-free human plasma and whole blood used for the preparation of the calibration standards and quality control (QC) samples and validation of the DBS method, respectively, were obtained from pooled plasma from healthy volunteers and whole blood from 1 volunteer in an ethylenediaminetetraacetic acid tube (Amsterdam University Medical Centers, University of Amsterdam, the Netherlands). For DBS analysis, the Whatman 903 paper was purchased from van der Most (Heerde, the Netherlands).

### Apparatus and LC-MS/MS Settings

The LC-MS/MS system, pumps, column oven, autosampler, degasser, and spectrometer used in this study were identical to those used in a previous study.^18^ As chromatographic data system, Analyst 1.7 (Sciex, Concord, ON, Canada) was used as the chromatographic data system.

A HyPURITY C18 HPLC (50 × 2.1 mm, 3.0 μm) column (Thermo Scientific, Waltham, MA) was used to separate the 7 analytes at 0.5 mL/min. The injection volumes were 2 μL for the plasma and 5 μL for the DBS samples. The mobile phase, gradient, and total run time of 6.5 minutes were similar to those of our previously described LC-MS/MS settings, as were the temperatures of the column and autosampler.^18^ The analytes were detected in the positive electrospray ionization mode. The ion spray voltage was set at 5500V. Mass transitions and collision energies of elexacaftor, elexacaftor-M23, and tezacaftor-M1 are shown in Table 1. The latter values for ivacaftor, ivacaftor carboxylate, hydroxymethyl ivacaftor, and tezacaftor can be found in a previous study.^18^

**TABLE 1.** Mass Transitions, Declustering Potential, Entrance Potential, Collision Cell-Exit Potential, and Collision Energy of Elexacaftor, Elexacaftor-M23, and Tezacaftor-M1

Analyte	Mass Transition		Declustering Potential (V)	Entrance Potential (V)	Collision Cell Exit Potential (V)	Collision Energy (V)
	Precursor (m/z)	Fragment (m/z)				
Elexacaftor	598.20	329.10	41	10	22	45
Elexacaftor-M23	584.23	502.20	116	10	10	27
Tezacaftor-M1	519.19	441.10	181	10	14	35

m/z, mass-to-charge-ratio; V, voltage.

### Preparation of Stock Solutions Calibration Standards, Internal Standards, and Control Samples

Stock solutions of each analyte were prepared in MeOH at a concentration of 1 mg/mL, and ivacaftor was prepared in MeOH/acetic acid (1:1). Subsequently, stock solutions were diluted in MeOH/H_2_O (1:1) to a concentration of 20 mg/L, except for tezacaftor-M1, which was diluted to 100 mg/L. Seven calibration standards for each analyte (0.01, 0.05, 0.1, 0.5, 1, 5, and 10 mg/L) were prepared in whole blood for DBS validation, except for tezacaftor-M1, for which calibration standards were prepared at 0.025, 0.125, 0.25, 1.25, 2.5, 5, and 10 mg/L. The concentration ranges were similar to those in plasma, except for the highest standard of tezacaftor-M1 which was 12.5 mg/L. For DBS calibration standards, 50 µL was spotted on the Whatman 903 paper and dried for 24 hours at room temperature. The stock solution and DBS calibration standards were stored at −20°C, and the plasma calibration standards were stored at −80°C until use. Stock solutions of the IS were prepared in MeOH at a concentration of 0.1 mg/mL. The IS working solution was prepared by diluting the IS stock solution in ACN:MeOH (420:80) to reach a concentration of 0.05 mg/L for ivacaftor-D9 and 0.15 mg/L for elexacaftor-D3 and tezacaftor-D4. The IS working solution was stored at −20°C until use. For each analyte, except for tezacaftor-M1, QC samples were prepared at 4 concentration levels on DBS and in human plasma: The predefined lower limit of quantification (QC LLOQ) at 0.01 mg/L, 3 times the QC LLOQ (QC LOW) at 0.03 mg/L, a middle concentration level (QC MLQ) at 0.5 mg/L, and a high concentration level (QC HLQ) at 7.5 mg/L. For tezacaftor-M1, QC samples were prepared on DBS and in human plasma: QC LLOQ, 0.025 mg/L; QC LOW, 0.075 mg/L; QC MLQ, 1.25 mg/L, and QC HLQ, 7.5 mg/L in DBS and plasma. For DBS QC samples, 50 µL was spotted on the Whatman 903 paper and dried for 24 hours at room temperature. The DBS QC samples were stored at −20°C, and QC samples in plasma were stored at −80°C until use.

### Pretreatment of the Samples

#### DBS

A DBS spot (8 mm) was punched out into a glass tube. Compounds were extracted by adding 250 μL of the IS solution to the samples (elexacaftor-D3 for elexacaftor and elexacaftor-M23, tezacaftor-D4 for ivacaftor, ivacaftor carboxylate, hydroxymethyl ivacaftor, tezacaftor, and tezacaftor-M1). Subsequently, ultrapure water (100 μL) was added to the mixture. The samples were vortexed for 5 minutes in a multitube vortex at maximum speed and then placed in an ultrasonic bath for 10 minutes. Then, 250 μL of the solution was pipetted into a 1.5 mL vial. Five μL of the supernatant was injected into the LC-MS/MS system.

#### Plasma

The pretreatment of the plasma samples was identical to our previously described method. Briefly, after thawing and vortexing, the plasma samples were precipitated with the IS solution before injection into the LC-MS/MS system.^18^

### Calculation of the Concentration

The MS response was expressed as the integrated area of the chromatographic peak. For calibration, the concentrations of the prepared calibration standards were known variables (*x*). The ratio of the analyte MS response to the IS MS response per calibration level was the unknown variable (*y*). Samples from the pwCF were back-calculated using the calibration line based on their respective area ratio of the analyte/IS MS response. For all components in DBS, a quadratic curve of 1/x fit best, and for all components in plasma, a quadratic curve of 1/x^2^ fit best. Calculation and reporting of the concentrations were performed automatically using Analyst 1.7.

### Validation of the DBS Method

Linearity, accuracy, precision, stability, hematocrit (Hct), spot-to-spot carryover, spot volume, extraction efficiency, and method comparison (clinical validation in patients: venous blood vs. capillary blood on DBS) were validated for all analytes. These parameters were based on a guideline for the validation of DBS methods for TDM purposes, which corresponds to the requirements of the FDA and EMA guidelines for bioanalytical method validation but is more extensive for DBS methods.^19–21^ Only the methods for the DBS-specific parameters (Hct, spot-to-spot carryover, spot volume, extraction efficiency, and method comparison) are described below. For the other parameters, the method can be found in our previous article.^18^

### Hematocrit

Hct influences the viscosity of blood and therefore spreads on the Whatman 903 paper. Whole blood samples obtained from different healthy volunteers were used to prepare the calibration standards and QC samples. The Hct values of the calibration standards and QC samples were 0.30 and 0.40, respectively. The measured concentrations of the QC and calibration standard samples should be to 85%–115%.

### Spot-to-Spot Carryover

The possibility of the carryover of analytes from 1 spot to a subsequent spot (during punching) was assessed by measuring an empty spot (as a blank) 6 times next to a QC HLQ spot. The average areas of the 6 blanks on the retention time of the analytes and IS were compared with the average areas of the 6 QCs LLOQ measured for accuracy and precision. The carryover of HLQ on the blank should not exceed 20% of the LLOQ of the analyte and not more than 5% of the IS.

### Spot Volume

To validate the differences in spot volume obtained in patient samples by fingerprick (which is an uncontrolled volume and will vary), 30, 50, and 70 μL of QC LOW and QC HLQ were spotted in triplicate on a Whatman 903 paper. A minimum spot volume of 30 μL was used for a puncher of 8 mm, which was the minimum spot volume required for a valid spot. DBS samples were pretreated as described above and their accuracy was determined. The measured concentration was between 85% and 115% of the nominal concentration (in a regular spot volume of 50 μL).

### Extraction Efficiency

The extraction efficiency was determined by spotting QC LOW and QC HLQ samples in triplicate on a Whatman 903 paper. The extraction procedure was followed, and the extraction efficiency was determined. The extraction efficiency was >80% with an RSD of <5%.

### Clinical Validation of the DBS Method: Subjects and Study Design

The local Medical Ethics Review Committee approved the study design for the clinical validation of DBS (ABR: NL70667.018.19). All participants provided written informed consent before enrollment in the study. In this prospective, observational, cohort, single-center study, pwCF (adults and children) were enrolled for the clinical validation of the DBS method, which was a substudy in a larger multicenter longitudinal study.^22^ PwCF were using ETI according to a compassionate use program because at that time, ETI was not yet registered in the Netherlands. No formal sample size calculations were performed; however, we aimed to collect a minimum of 20 paired DBS plasma samples. Venous blood and DBS were subsequently sampled via fingerprick (max. Ten min between samples) at random time points after ETI administration. If a subject was sampled more than once, the time interval between visits was approximately 3 months. When a patient was sampled for the first time, the DBS sample was collected by a trained health care professional. Subsequently, DBS samples were collected from the patients. The fingerprick was performed using a contact-activated lancet (BD Microtainer 2.0 mm × 1.5 mm). Two spots of blood were collected on the Whatman 903 paper. After collection, samples were dried for a minimum of 30 minutes at room temperature. After drying, the samples were packed in plastic zip-lock bags and stored in a freezer (−20°C) until analysis. All samples were pretreated as described above, and their concentrations were measured.

### Clinical Validation of the DBS Method—Method Comparison: Venous Blood Versus Capillary Blood on DBS

Blood obtained with a fingerprick (capillary) often differs from the plasma generated from venous blood.^21^ To compare the 2 sampling methods, the following steps were followed. First, the Passing–Bablok regression analysis was performed to calculate the intercept and slope of the linear regression to assess proportional and constant bias, respectively.^21^ Then, Passing–Bablok regression equations were used to calculate the “estimated plasma concentration” (EPC) from the measured DBS concentration by equation 1.

(1) EPC=slope×[DBS]+intercept.

Subsequently, a Bland–Altman plot was made to evaluate the mean difference between the EPC and plasma concentrations (bias) and the limits of agreement (±1.96 SD).^21^

According to the EMA guideline on bioanalytical method validation, the acceptance criterion for cross-validation was followed, where for ≥67% of the samples, the difference between EPC and plasma should be within 20% of the mean of these values.^20^ The acceptance value was calculated using equation 2.

(2) $$\text{difference}\left(\%\right)=\frac{\left[\text{EPC}\right]-\left[\text{plasma}\right]}{\text{plasma}}\times 100\%.$$

Finally, the predictive performance was evaluated using the jackknife method, as only a limited number of paired samples were available. With this method, the original dataset with n (=21) samples was resampled n−1 (=20) times, creating all different subsets leaving 1 sample out.^21^ Each subset was used to formulate an equation by the Passing–Bablok regression, which was used to calculate the EPC of the left-out sample. Bias was assessed using the median percentage predictive error (MPPE) (equation 3) and precision was assessed using the median absolute percentage predictive error (MAPE) (equation 4).^23^

(3) $$\text{MPPE}=\text{median}\left(\frac{\left[\text{EPC}\right]-\left[\text{plasma}\right]}{\left[\text{plasma}\right]}\right)\times 100\%.$$

(4) $$\text{MAPE}=\text{median}(|\frac{\left[\text{EPC}\right]-\left[\text{plasma}\right]}{\left[\text{plasma}\right]}|)\times 100\%$$

Acceptance limits of ≤15% for MPPE and MAPE were defined, in line with other studies.^24,25^ Statistical analysis was performed using R (version 4.3.1, Vienna, Austria) and Microsoft Office Excel (Microsoft, Redmond, WA).

### Validation Method in Plasma

For the 3 new analytes (elexacaftor, elexacaftor M-23, and tezacaftor-M1), the following parameters were validated in plasma according to the requirements of the EMA guideline for bioanalytical method validation^26^: Selectivity, carryover, linearity, accuracy and precision, dilution, matrix effect, and stability. In our previous study, the validation method in plasma was described for ivacaftor, its metabolites, lumacaftor, and tezacaftor and was identical for the new analytes.^18^

## RESULTS

### Chromatography

**FIGURE 2.**
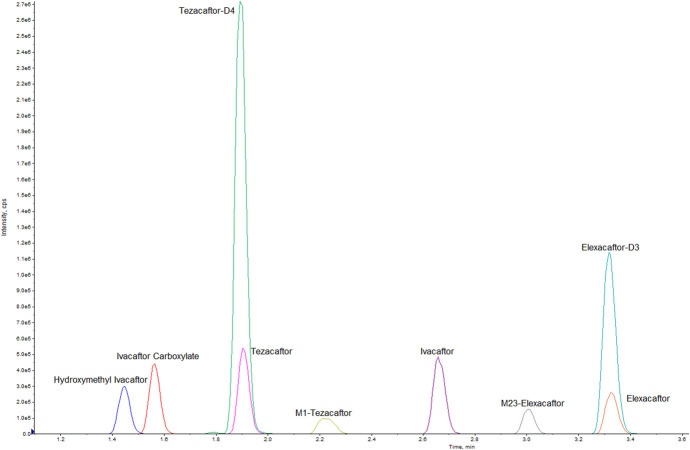
Chromatogram of the 9 analytes of concentration level MLQ in DBS: hydroxymethyl ivacaftor, ivacaftor carboxylate, tezacaftor, tezacaftor-D4 (IS), tezacaftor-M1, ivacaftor, elexacaftor-M23, elexacaftor-D3 (IS), and elexacaftor. MLQ—middle concentration level; DBS—dried blood spot.

Ivacaftor, ivacaftor carboxylate, hydroxymethyl ivacaftor, tezacaftor, tezacaftor-M1, elexacaftor, elexacaftor-M23, tezacaftor-D4, and elexacaftor-D3 were successfully analyzed in 1 run, and the total run time was 6.5 minutes. The ion chromatogram is shown in Figure 2.

### Validation in DBS

The validation parameters for DBS are listed in Table 2. The preset requirements for within-run and between-run accuracy and precision were met. Puncher carryover was observed because spots which were punched after the QC HLQ exhibited higher concentrations. To correct for this, a new validation parameter was developed: Puncher carryover, in which a ULOQ was punched, and 3 blanks were subsequently added. This prevented carryover by the puncher and negated the effect. Therefore, after a high calibration standard, QC HLQ, and every patient sample, 3 blanks were punched (but not further analyzed). Spot volume had no influence on the measured concentrations of all analytes because for both low (30 µL) and high (70 µL) spot volumes, the measured concentrations fell within 85%–115% of the nominal concentrations. All analytes were extracted successfully from the Whatman 903 paper by the described extraction method, as the extraction efficiency was >80% for all analytes. Calibration standards, quality controls, and freezer stability samples were prepared from fresh whole blood of healthy volunteers. The Hct values of the calibration standards (0.30 L/L) and the quality controls (0.40 L/L) differed significantly. The quality controls were within ±15% of the nominal concentration, so no Hct effect was observed. Samples were stable for at least 5 months in the freezer (−20°C), 96 hours at room temperature, and 96 hours in the autosampler (10°C).

**TABLE 2.** Overview of Validation Parameters of Ivacaftor, Ivacaftor-M1, Ivacaftor-M6, Tezacaftor, Tezacaftor-M1, Elexacaftor, and Elexacaftor-M23 in DBS

		Analytes						
		Parameter	Level	IVA	IVA-M1	IVA-M6	TEZ	TEZ-M1	ELX	ELX-M23
Accuracy (%) (n = 6)	LLOQ	112.8	104.3	108.9	110.7	104.5	105.8	108.2
	LOW	99.5	99.6	93.9	95.8	97.7	96.1	99.2
	MLQ	89.5	90.5	89.1	88.3	89.7	89.8	89.7
	HLQ	99.1	99.1	85.3	87.7	96.7	88.7	95.7
Within-run precision CV (%) (n = 6)	LLOQ	5.1	5.2	5.3	2.9	5.7	3.2	15.0
	LOW	2.5	3.9	1.9	2.2	2.8	2.6	13.7
	MLQ	5.6	4.6	6.4	4.8	5.3	5.1	8.8
	HLQ	2.0	2.1	2.6	3.3	3.0	2.2	4.0
Between-run precision CV (%) (n = 6)	LLOQ	11.8	15.5	12.2	7.5	6.7	10.9	6.1
	LOW	9.6	4.5	6.0	2.5	10.7	2.6	2.6
	MLQ	10.1	7.0	9.9	9.1	8.7	8.9	5.5
	HLQ	3.1	2.6	3.2	5.7	5.5	3.5	2.4
Spot-to-spot carryover (%)		2.0	1.7	1.7	4.2	1.0	0.0	2.6
Spot volume 30 µL*	LOW	1.2 ± 6.2	−0.6 ± 4.0	1.8 ± 3.5	−1.7 ± 2.2	−3.6 ± 1.1	0.1 ± 1.5	−2.1 ± 2.0
	HLQ	−8.3 ± 0.5	−8.3 ± 2.2	−8.0 ± 1.3	−7.7 ± 2.9	−9.8 ± 2.7	−2.6 ± 1.4	−10.8 ± 3.2
Spot volume 70 µL*	LOW	−3.9 ± 1.6	−4.8 ± 2.5	−5.3 ± 2.8	−4.7 ± 2.1	−7.1 ± 4.3	−11.3 ± 2.7	−5.7 ± 1.8
	HLQ	−8.5 ± 4.5	−10.0 ± 5.8	−12.0 ± 6.7	−9.7 ± 4.6	−11.2 ± 4.6	−10.8 ± 6.8	−10.5 ± 7.0
Extraction efficiency (%)	LOW	−18.9	3.7	2.2	−1.3	−16.1	−3.9	−5.9
	HLQ	−8.2	−1.0	4.2	−6.6	−12.5	−4.7	−4.1
Freezer (−20°C) 5 months stability*	LLOQ	−6.3 ± 2.8	−2.0 ± 5.6	−15.6 ± 3.5	−7.2 ± 4.7	0.6 ± 5.2	−5.3 ± 3.6	−9.5 ± 3.0
	MLQ	−9.7 ± 6.9	3.5 ± 6.1	−14.7 ± 1.8	−8.3 ± 7.6	−13.5 ±	−14.9 ± 5.4	−10.1 ± 6.6
Room temp. 96 h stability*	LOW	15 ± 3.4	−2.6 ± 3.2	−8.2 ± 4.9	0.6 ± 1.6	7.3 ± 2.2	1.0 ± 2.9	14.9 ± 3.1
	HLQ	0.0 ± 4.6	7.5 ± 3.5	−4.4 ± 4.4	−0.5 ± 3.7	9.8 ± 4.6	−1.6 ± 2.3	−1.4 ± 3.1
Autosampler (10°C) 96 h stability (%)	LOW	−1.0	6.3	5.0	−4.0	−0.7	0.0	1.0
	HLQ	2.0	2.9	−6.7	−4.9	3.3	−8.3	1.2

* Data are presented as percentage deviation of the measured concentration versus the nominal concentration ± CV (%) (n = 3).

QC levels: HLQ, high concentration level; LLOQ, lower limit of quantification; LOW, 3 times the LLOQ; MLQ, middle concentration level.

### Clinical Validation of the DBS Method

Twenty-one paired DBS and plasma samples were collected from 12 pwCF. In Table 3, the baseline characteristics, Hct values, and concentration ranges of the analytes in the plasma and DBS are shown. The concentrations measured in the DBS were lower than those measured in the venous plasma. To account for this difference, statistical analysis was performed as described below.

**TABLE 3.** Baseline Characteristics

Baseline Characteristics	n = 12
Age (yr)	27.5 (13–52)
Sex, N (%)	
Female	8 (67%)
Weight (kg)	59.4 (29.7–75.2)
Daily dose (mg)	
Elexacaftor	200
Tezacaftor	100
Ivacaftor	300 (150 BID)
Total number of paired samples	21
Number of paired samples per patient	1.5 (1–3)
Hematocrit (L/L)	0.34 (0.26–0.40)
Concentration range in venous plasma (mg/L)	
Elexacaftor	9.52 (2.55–18.5)
Elexacaftor-M23	4.49 (1.35–10.8)
Tezacaftor	4.33 (1.25–10.7)
Tezacaftor-M1	6.10 (3.69–7.60)
Ivacaftor	1.62 (0.694–4.41)
Ivacaftor carboxylate	1.82 (0.606–3.91)
Hydroxymethyl ivacaftor	2.33 (1.04–5.32)
Concentration range in DBS (mg/L)	
Elexacaftor	5.33 (1.57–9.99)
Elexacaftor-M23	2.65 (0.865–7.00)
Tezacaftor	2.57 (0.65–5.98)
Tezacaftor-M1	4.29 (2.29–5.70)
Ivacaftor	0.800 (0.294–2.10)
Ivacaftor carboxylate	1.07 (0.351–2.34)
Hydroxymethyl ivacaftor	1.41 (0.617–3.09)

Numbers presented as median (range) or number (%).

The slope and intercepts of the Passing–Bablok regression are shown in Table 4 (see **Supplemental Digital Content**, **Figures 1–7**, http://links.lww.com/TDM/A771). A proportional bias was observed for all analytes except tezacaftor-M1, as the 95% confidence interval of the slope did not include 1. For tezacaftor-M1, a constant bias was observed as the 95% confidence interval of the intercept did not include a 0. Corrective actions were taken to account for the observed bias, and 3 different correction factors were assessed to transform the DBS concentration into venous plasma concentrations: Correction by (1) measuring the Hct value in the sample, (2) a constant plasma/DBS factor, and (3) the Passing–Bablok regression equation. The latter correction best described the agreement between DBS and the corresponding venous plasma concentrations as determined by the acceptance value (equation 2).

**TABLE 4.** Passing–Bablok Regression, Acceptance Values, and Predictive Performance Results

Analyte	Passing–Bablok		Acceptance Value		Predictive Performance	
	Slope (95% CI)	Intercept (95% CI)	Within 20% of Mean (%)	Within 15% of Mean (%)	MPPE (%)	MAPE (%)
Elexacaftor	1.93 (1.79–2.12)	−0.86 (−1.88 to 0.0043)	100	95	−0.48	7.04
Elexacaftor-M23	1.64 (1.54–1.80)	−0.23 (−0.71 to 0.023)	100	95	−0.41	4.61
Tezacaftor	1.66 (1.59–1.75)	0.23 (0.0052–0.45)	100	100	−0.085	4.85
Tezacaftor-M1	1.08 (0.92–1.26)	1.49 (0.75–2.09)	100	100	−0.39	4.31
Ivacaftor	1.95 (1.56–2.23)	0.057 (−0.065 to 0.33)	71	57	−0.050	13.64
Ivacaftor carboxylate-M6	1.83 (1.58–2.14)	−0.23 (−0.55 to 0.067)	90	81	1.76	10.23
Hydroxymethyl ivacaftor-M1	1.63 (1.29–2.04)	0.032 (−0.65 to 0.44)	81	62	−0.85	15.05

Acceptance values of differences between EPC and plasma values within 20% and 15% of the mean. Predictive performance by the jackknife method.

The Bland–Altman analysis showed good agreement between the EPC and plasma values (see **Supplemental Digital Content**, **Figures 8–14**, http://links.lww.com/TDM/A771). No fixed bias was observed, as all 95% CIs of the mean included 0%, and 95% of the differences were within the 1.96 SD limits. For all the analytes, the acceptance values were within 20% of the mean for a minimum of 67% of the repeats, according to the EMA guidelines (Table 4).^20^ For elexacaftor, elexacaftor-M23, tezacaftor, and tezacaftor-M1, higher acceptance values were met, as, respectively, 95%, 95%, 100%, and 100% of the repeats were within 15% of the mean. The predictive performance of the conversion equations was good for all the analytes, as the MPPE and MAPE were within the acceptance limits of <15%. Except for hydroxymethyl ivacaftor, MAPE (15.05) exceeded this limit.

### Validation of Additional Analytes in Plasma

The plasma validation results of the new analytes elexacaftor, elexacaftor-M23, and tezacaftor-M1 are described in the **Supplemental Digital Content 1** (see http://links.lww.com/TDM/A771).

## DISCUSSION

In this study, we developed and validated a DBS method for simultaneous quantification of the most commonly prescribed CFTR modulators and their metabolites. Our previously described LC-MS/MS method for the quantification of lumacaftor, tezacaftor, ivacaftor, ivacaftor carboxylate, and hydroxymethyl ivacaftor in plasma was used and expanded with elexacaftor, tezacaftor-M1, and elexacaftor-M23. Moreover, the development and validation of DBS as a new matrix have been added.

In the literature, there are other LC-MS/MS methods describing the quantification of the CFTR-modulating drugs, including the most recent modulator elexacaftor. However, none of these published methods describe the quantification of the active metabolite elexacaftor-M23, and the validated matrix is limited to plasma or serum.^27–29^

We encountered several challenges during method development and validation. In this DBS method, tezacaftor-D4 was used as an IS for ivacaftor, ivacaftor carboxylate, and hydroxymethyl ivacaftor because during stability studies at room temperature, the area of the IS ivacaftor-13C6 and ivacaftor-D19 decreased. Hence, we decided to exclude ivacaftor IS during validation and used tezacaftor-D4 instead. Tezacaftor-D4 was the best IS candidate because it eluted between ivacaftor and its metabolites. Additionally, during validation, puncher carryover was observed for CFTR-modulating drugs. According to Capiau et al,^21^ no puncher-mediated carryover has been described for therapeutic drugs, and a literature search revealed no findings in this area. Puncher carryover was validated as an additional parameter and was easily invalidated by punching 3 blanks after the highest QC level, highest calibration standard, and patient samples. Next, according to the FDA guideline, the “homogeneity of sample spotting” or in Capiau et al described as “the volcano effect” should be evaluated as a DBS-specific parameter.^19,21^ However, in our method, a large puncher with a diameter of 8 mm was used, which punches almost the entire spot (diameter of 10 mm). Therefore, the variation in punch location was negligible, and the volcano effect was not evaluated. Lastly, the volume fraction of blood taken by red blood cells (Hct) can significantly affect the recovery of the drug of interest. Therefore, different levels of Hct should be evaluated. During validation, we demonstrated that Hct had no influence on the recovery of CFTR modulators and related metabolites. This was also observed in the clinical validation, where the correction for Hct bias was insignificant. Hct levels in the pwCF samples were comparable with the validated Hct levels. As described in the literature, PwCF tend to have Hct levels comparable with those in the regular population.^30^

The strength of this study is that it is the first to describe the development and successful clinical validation of a DBS method for quantifying CFTR modulators. Another strength is that all CFTR modulators and their main metabolites can be measured in 1 run of only 6.5 minutes, which enables the high-throughput analysis of samples. Also, to the best of our knowledge, the quantification of elexacaftor-M23 has not been described before. Elexacaftor-M23 is the main metabolite of elexacaftor which is pharmacologically active and has comparable potency.^14^ Furthermore, for the clinical validation of the DBS method, the EMA criterion for method comparison was met for all analytes, as >67% of the samples were within 20% of the mean difference.^20^ For elexacaftor, tezacaftor, and their metabolites, even higher acceptance criteria were met, as all recalculated DBS concentrations and matched venous plasma samples were within 15% of the mean for >95% of the repeats. Finally, the predictive performance of the conversion of DBS concentration into EPC was good, as the MPPEs and MAPEs were within the acceptance limit of <15%. Only hydroxymethyl ivacaftor showed moderate predictive performance, with a MAPE of 15.05%.

The limitations of this study include the small sample size for the clinical validation of the DBS method. The guidelines for measurement procedure comparison by the Clinical and Laboratory Standards Institute (CLSI) state that a minimum of 40 paired patient samples should be analyzed during clinical validation.^31^ The study was performed when the number of pwCF undergoing ETI was still limited, as the clinical validation of the DBS method was added to an ongoing clinical trial where only 20 pwCF were using ETI on a compassionate use basis. The trial was conducted during the COVID-19 pandemic, so many visits to this vulnerable patient group were canceled, and it was not possible to match venous and DBS samples from every patient. Therefore, it was decided to sample the same participant more than once, as visits were separated by approximately 3 months, resulting in 21 paired DBS and plasma samples. However, as stated above, the predictive performance evaluated using the jackknife method was good. The jackknife method was used because only a limited number of paired samples were available.^21^ Moreover, the EMA criteria for method comparison were met for all analytes; however, stricter criteria were applied for validation studies in clinical practice. However, with the limited data available and the proposed use of this method, we can now accept this limitation. TDM of CFTR modulators has not yet been implemented in clinical practice because good reference values and exposure–response relationships are lacking.

## CONCLUSIONS

In conclusion, a LC-MS/MS quantification method for the analysis of elexacaftor, elexacaftor-M23, tezacaftor, tezacaftor-M1, ivacaftor, ivacaftor carboxylate, and hydroxymethyl ivacaftor in DBS was developed and clinically validated. Next, the already existing method in plasma was expanded with elexacaftor, elexacaftor-M23, and tezacaftor-M1. Currently, ETI is considered the most effective CFTR-modulating drug and is widely applied. However, in real-life studies, a considerable number of patients show a lack of effects or side effects during treatment, which may be partly explained by the variance in drug exposure. Our methods allow for the quick and convenient determination of drug concentrations and may therefore be used both in clinical care and research to address unanswered PK questions.
